# A dual-view videos and two-dimensional pose annotation dataset of Taekwondo Poomsae (Taegeuk 1–8) for human motion analysis

**DOI:** 10.1016/j.dib.2026.113324

**Published:** 2026-10-09

**Authors:** Chien-Chung Huang, Qing-Wei Zheng

**Affiliations:** a Department of Recreation Management**,** Shih Chien University Kaohsiung Campus**,** Taiwan; b Department of Computer Science & Information Engineering**,** National Kaohsiung University of Science and Technology**,** Kaohsiung**,** Taiwan

**Keywords:** Human pose estimation, Motion analysis, High-speed movement, 2D skeletal annotation, Taekwondo Poomsae, Sports technology, Dual-view dataset

## Abstract

Computer vision in sports increasingly supports judging, analytics, training, and injury prevention, yet Taekwondo’s high-speed kicks introduce motion blur, self-occlusion, and large joint excursions that challenge models trained on everyday actions. We release a synchronized dual-view dataset of standardized Taekwondo Poomsae (Taegeuk 1–8), recorded concurrently from frontal and lateral viewpoints in Full HD (1920 × 1080, 30 fps). Each frame includes normalized 2D coordinates and confidence scores for 12 major joints (shoulders, elbows, wrists, hips, knees, ankles), distributed as frame-aligned CSV files paired with the videos. Dual viewpoints provide complementary evidence for fast, large-amplitude lower-limb motion, enabling robust 2D pose model training/evaluation, cross-view generalization tests (e.g., train-frontal/test-lateral), and reproducible benchmarks for sports motion analysis. This resource addresses the scarcity of sports-specific, high-speed data and supports fair, replicable evaluation pipelines in computer vision.

Specifications Table

**Table:** 

Subject	Computer Sciences
Specific subject area	High-speed human motion analysis and 2D pose estimation in Taekwondo Poomsae, utilizing synchronized dual-view video recordings (frontal and lateral perspectives).
Type of data	Video recordings and numeric tabular data (CSV files containing frame-by-frame 2D joint coordinates and confidence scores).
Data collection	The dataset was acquired using two fixed cameras (Insta360, 1080p, 30 fps) mounted on tripods to synchronously record Taekwondo Poomsae (Taegeuk 1–8) from frontal and lateral views; a custom trigger simultaneously started/stopped both cameras, keeping inter-view timing within ±1 frame (∼33 ms). For each form, complete performances were captured from both views; when multiple takes existed, the take with no occlusion, complete execution, and acceptable motion blur was selected. Videos were then annotated frame-by-frame for 12 major joints (shoulders, elbows, wrists, hips, knees, ankles), yielding 2D coordinates and detection confidence per joint; coordinates were normalized to the frame (x,y ∈ [0,1]) with no temporal smoothing applied, and undetected joints were recorded with NaN coordinates and a confidence of 0.0.
Data source location	Sanmin District, Kaohsiung City, Taiwan — Basement Taekwondo training room, Kaohsiung Municipal Zhengxing Junior High School.
Data accessibility	Repository name: Mendeley Data Data identification number: 10.17632/bjy7vr4xkt.1 Direct URL to data: https://data.mendeley.com/datasets/bjy7vr4xkt/1
Related research article	*None*

## 1 Value of the Data

• **Model training and evaluation (2D):** Suitable for training/evaluating 2D pose estimation and action classification models, supplementing the scarcity of high-speed, large-amplitude samples in existing public datasets.
• **Viewpoint-robustness testing:** Enables cross-view assessment (e.g., train on frontal, test on lateral) to quantify model sensitivity to viewpoint changes.
• **Motion-consistency quantification:** Under frame-aligned conditions, dual-view skeletons allow comparison of the same form across views or repeated trials; 2D metrics (e.g., joint-angle variation, DTW distance) can be used as reference measures.
• **Benchmarking and reproducibility:** Provide a fixed-column wide-format CSV, one-to-one video–annotation pairing, and clear naming/directory conventions, facilitating reproducible train/test pipelines and method comparisons.
• **Taekwondo motion analysis and sports science applications:** Taekwondo Poomsae consists of standardized and continuous offensive and defensive movements involving stepping, changes in body direction, upper-limb techniques, and lower-limb kicks. The dataset can support motion performance analysis, comparison of movement techniques, and sports training research in Taekwondo, providing a useful data resource for related studies in sports science and sports technology.

## 2 Background

In recent years, with the advancement of computer vision and deep learning methods, the application of related technologies in sports has become increasingly widespread. Image recognition and pose estimation have been employed in referee decision-making, competition analysis, and daily training, offering both real-time feedback and long-term monitoring of athlete performance [[1], [2], [3], [4], [5]]. These technologies not only assist referees in making more accurate judgments but also provide spectators with more intuitive insights into competitions, while simultaneously delivering athletes objective, data-driven evidence that supports performance improvement and helps prevent sports injuries caused by improper posture [6,7]. Collectively, such applications further promote the development of sports science.

The analysis of high-speed movements (e.g., Taekwondo kicking) presents challenges for computer vision: rapid displacements often cause motion blur and occlusions, while the large range of motion and high angular velocities exceed those of common daily activities. As a result, datasets and models constructed under everyday scenarios often suffer from domain gaps and performance degradation in this context [8]. Taekwondo Poomsae (Taegeuk 1–8) consists of standardized offensive and defensive sequences and is a common training component. Prior studies have examined interventions on core stability and proprioception in Poomsae athletes, demonstrating distinctive training and physical demands [9,10]. Within this framework, the construction of a corresponding machine vision-based skeletal annotation dataset carries both practical and research value.

To support model training and evaluation in high-speed motion scenarios, we collected and released a synchronized dual-view Taekwondo Poomsae dataset. The dataset includes frontal and lateral videos (1920 × 1080, 30 fps) of Taegeuk 1–8 sequences, along with corresponding frame-by-frame 2D joint annotations. The synchronization of two views provides complementary observations, mitigating the information loss caused by occlusion or limited visibility in single-view recordings [11,12]. The dataset covers a wide range of lower-limb kicking techniques with large motion amplitudes, producing joint sequence samples suited to high-speed and large-amplitude scenarios. This dataset can serve as training material, a benchmark for cross-view robustness evaluation, and a resource for motion consistency analysis. The file structure and naming conventions are detailed in the sections Data Description and Annotation File Information.

## 3 Data Description

This dataset provides frame-by-frame human pose annotations for Taekwondo Poomsae (Taegeuk 1–8), with each sequence corresponding to a single form and a single viewpoint.

### 3.1 Video specifications

Table 1 summarizes the acquisition properties of the raw video sequences. All videos were recorded in Full HD (1920 × 1080) at a fixed 30 fps, stored as .mp4 files using H.264/AVC1 encoding. Each file contains one complete performance of a Taekwondo Poomsae (Taegeuk 1–8), with paired recordings captured simultaneously from frontal and lateral viewpoints. This configuration ensures temporal consistency (inter-frame interval ≈ 33.333 ms) and broad compatibility with common media players.

**Table 1:** Video specification.

Item	Specification	Notes
Format	.mp4 (H.264/AVC1)	Compatible with standard media players
Resolution	1920 × 1080 pixels	Fixed Full HD
Frame rate	30 frames/s	Constant frame interval ≈ 33.333 ms
Viewpoints	Frontal and Lateral	Two fixed camera positions
Duration	Varies by sequence	One full performance per file

### 3.2 Annotation file specifications

Table 2 summarizes the structural details of the annotation files, which are released in .csv format (UTF-8 encoded). Each row corresponds to a single video frame and contains the frame index, the timestamp in milliseconds, and the normalized coordinates (x, y) with detection confidence values for 12 body joints. The ordering of joints is fixed to ensure consistency in parsing and reproducibility across sequences. The normalized coordinate system (x, y ∈ [0,1]) facilitates comparison across videos, while a pixel conversion formula (x1920, y1080) is also provided to enable direct alignment with the original frames.

**Table 2:** Summary of CSV annotation structure.

Item	Specification	Notes
Format	.csv (UTF-8 encoding)	Wide format representation
Granularity	One row = one frame	Strict alignment with video frames
Fields	frame_index, time_ms, 12 joints × (x, y, score)	Fixed joint order for parsinginterval ≈ 33.333 ms
Joint order	L_shoulder, R_shoulder, L_elbow, R_elbow, L_wrist, R_wrist, L_hip, R_hip, L_knee, R_knee, L_ankle, R_ankle	Consistent across all files
Coordinate system	Normalized (x, y ∈ [0,1])	Origin at top-left; pixel conversion x1920, y1080
Confidence score	Float [0,1]	Missing joints: (NaN, NaN, 0.0)

Each video frame provides the two-dimensional coordinates and detection confidence scores for 12 body joints, following a fixed joint order to facilitate parsing, validation, and model training. The dataset encompasses two standard camera perspectives: frontal and lateral (Fig. 1).

**Fig. 1:**
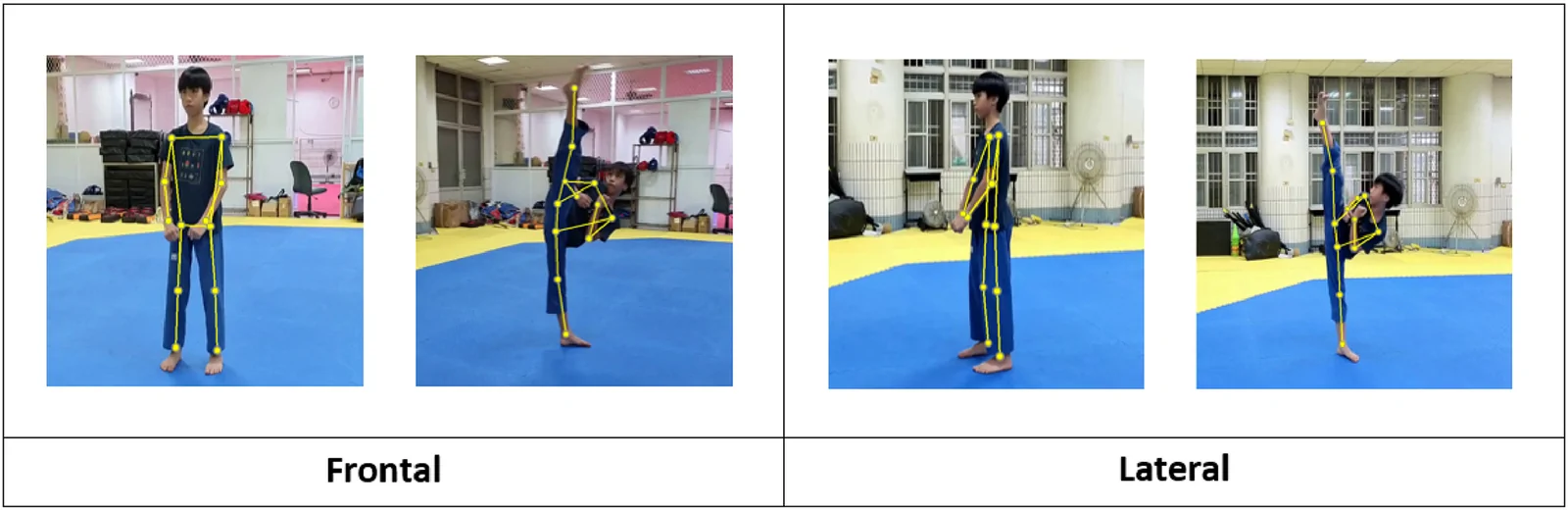
Frontal and lateral view illustration.

### 3.3 Data structure and naming conventions

The dataset is organized into two main directories: DATA/annotations and DATA/video. Each chapter (Taegeuk_<ordinal>) is further divided by perspective into Frontal and Lateral*.* For a given chapter and perspective, the video and its corresponding CSV file share the same base filename (e.g., 1st_Frontal.mp4… ⇄ 1st_Frontal.csv…) (Fig. 2).

**Fig. 2:**
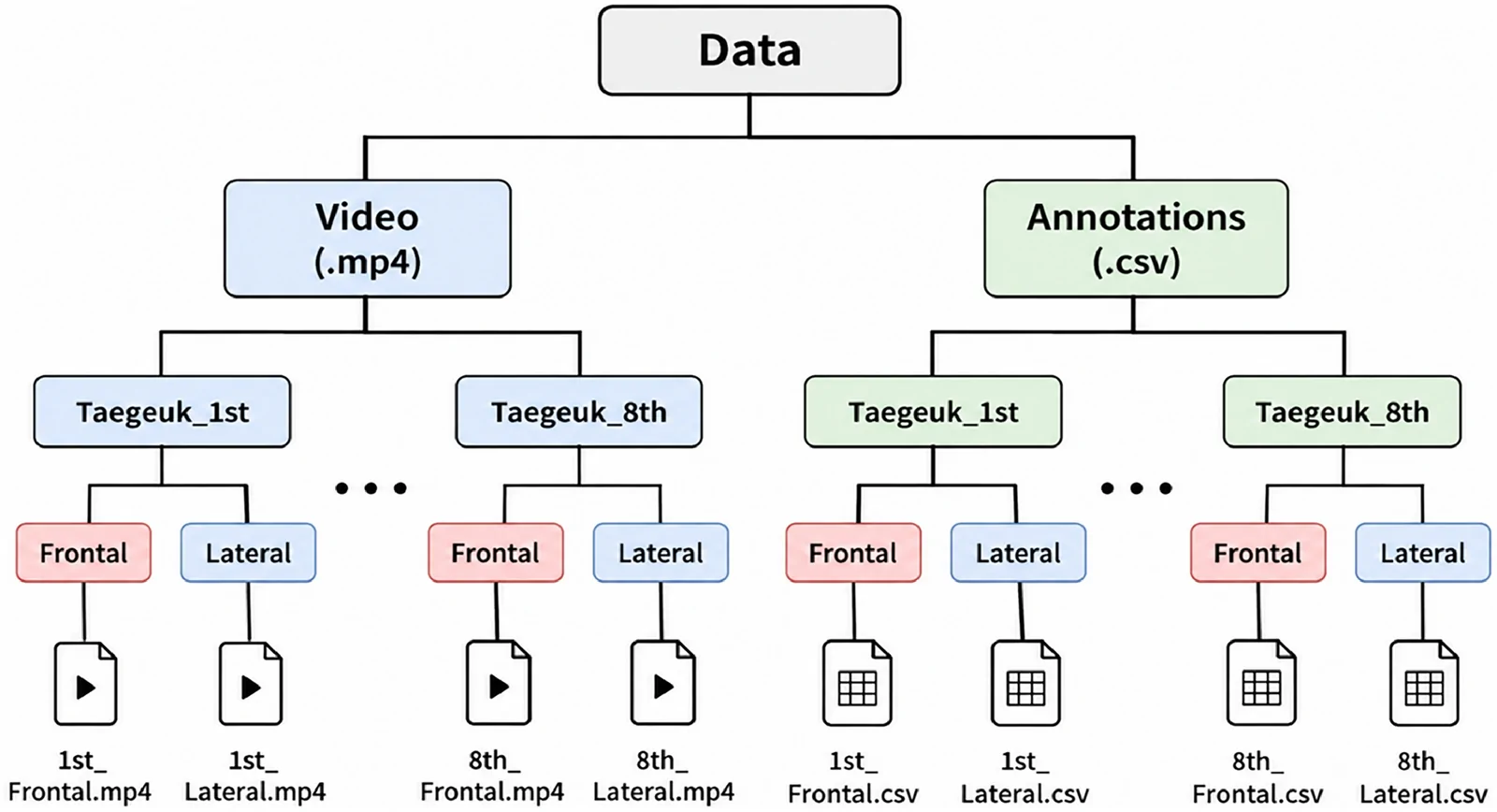
Schematic od dataset specifications.

## 4 Annotation File Information

### 4.1 File organization and granularity

Annotations are provided in CSV format (UTF-8), represented in wide format, where each row corresponds to a single video frame. Each CSV file is paired with its source video using the same base filename (e.g., 1st_Frontal.mp4… ⇄ 1st_Frontal.csv…). Rows are ordered sequentially by frame index and temporally aligned.

### 4.2 Column structure (left to right, fixed order)

• frame_index — Frame index, integer, starting from 0 (Fig. 3).

**Fig. 3:**
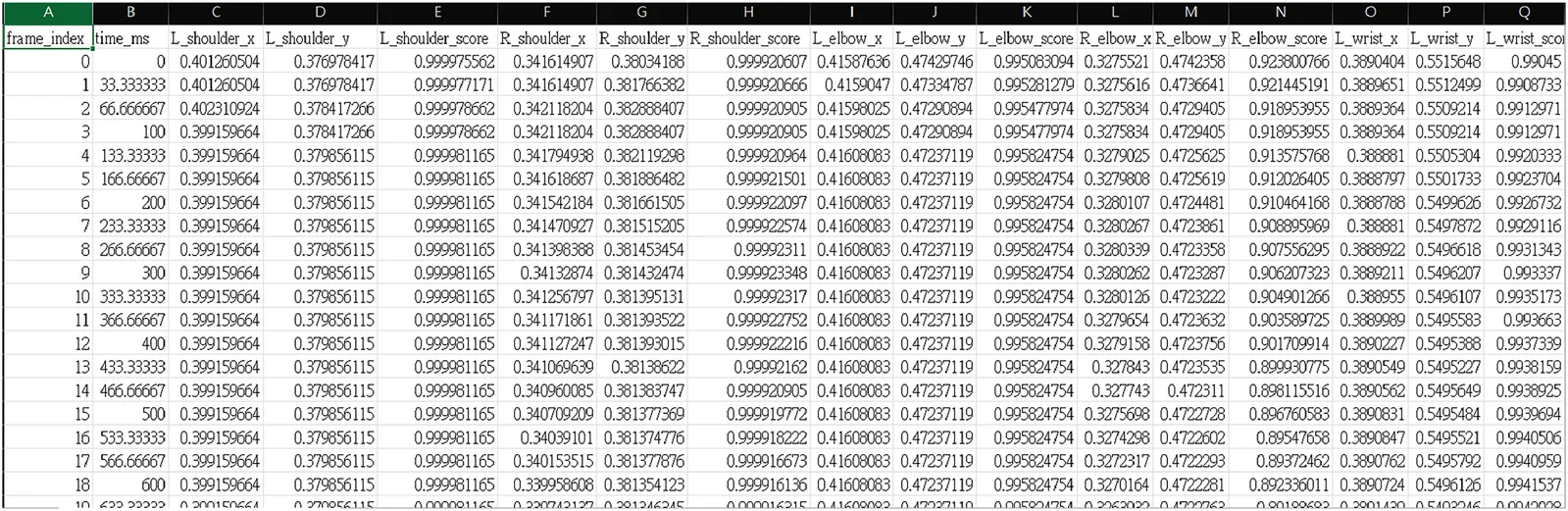
Example of CSV annotation file.
• time_ms — Frame timestamp in float (milliseconds); under 30 fps, the theoretical increment is 33.333… ms (Fig. 3).
• Repeated three-column groups for each joint: <joint>_x, <joint>_y, <joint>_score. The fixed joint order is
• L_shoulder, R_shoulder, L_elbow, R_elbow, L_wrist, R_wrist, L_hip, R_hip, L_knee, R_knee, L_ankle, R_ankle (Fig. 4).

**Fig. 4:**
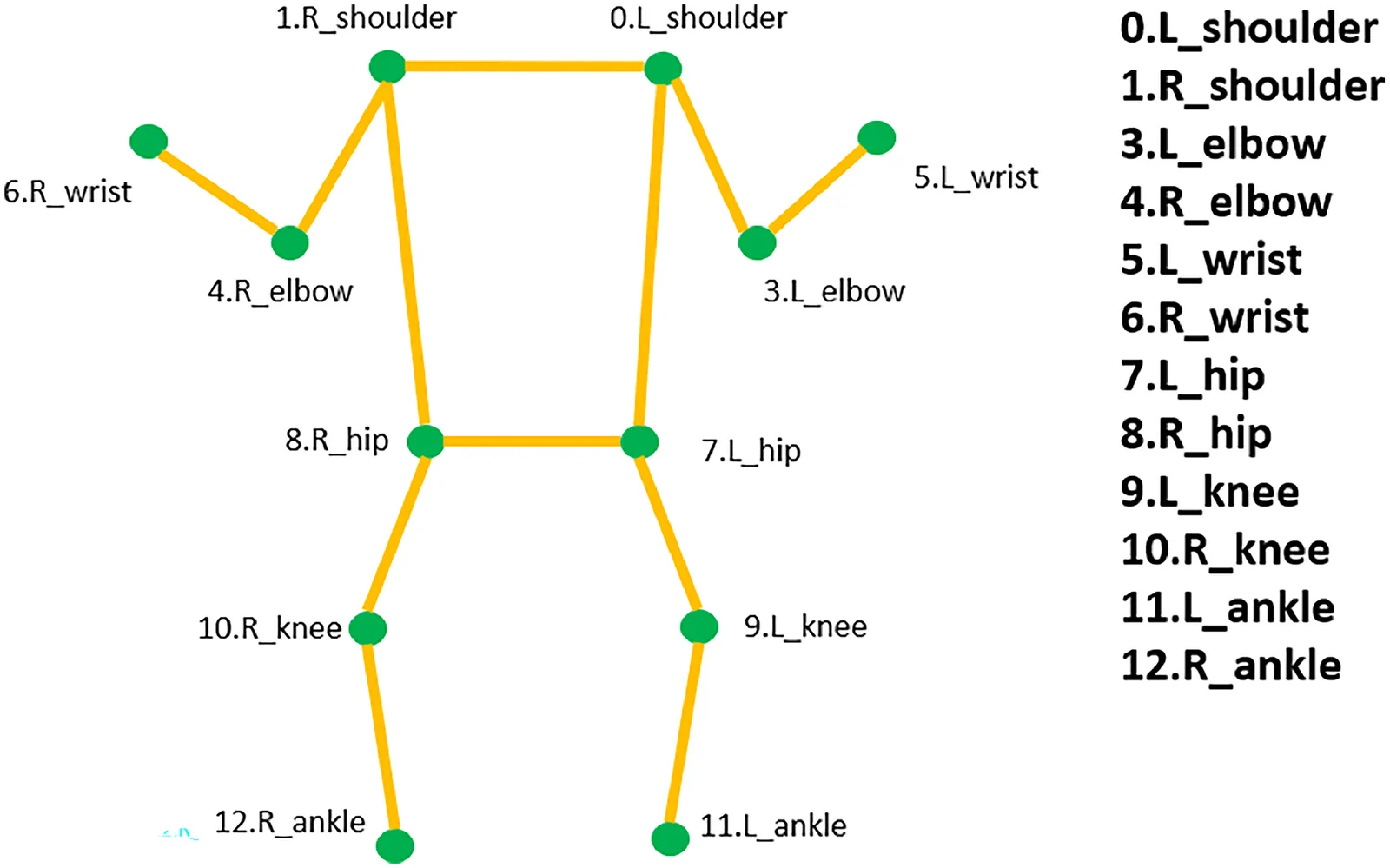
Joint name and location mapping.

### 4.3 Numerical semantics and conventions

Coordinates (<joint>_x, <joint>_y) are stored as float values, normalized relative to the frame dimensions; the theoretical range is [0,1]. In rare cases where out-of-bound values occur, it is recommended to clip them to [0,1] during downstream processing. Pixel conversion can be obtained as: x_px = x × 1920, y_px = y × 1080.

The Confidence (<joint>_score) is a float within [0,1], representing the detection confidence of the corresponding joint in a given frame.

The frame_index is recommended to be stored as int32; time_ms as float64, to minimize cumulative error in long sequences (Table 3).

**Table 3:** Field-level specifications of the annotation CSV file.

Field	Type	Range / Unit	Meaning	Notes
frame_index	int32	—	Frame index, starting at 0; monotonically increasing	Store as int32 for long-clip efficiency
time_ms	float64	milliseconds (ms)	Frame timestamp; theoretically ≈ frame_index × 1000/30	Fixed Full HD
<joint>_x	float32	[0, 1] (normalized width)	Joint x-coordinate (0 = left edge, 1 = right edge)	Pixel conversion x_px = x × 1920; rare out-of-range values should be clipped to [0,1]
<joint>_y	float32	[0, 1] (normalized height)	Joint y-coordinate (0 = top edge, 1 = bottom edge)	Pixel conversion y_px = y × 1080; rare out-of-range values should be clipped to [0,1]
<joint>_score	float32	[0,1]	Detection confidence for the joint in that frame	Occlusion/missing: x = NaN, y = NaN, score = 0.0

### 4.4 Annotation procedure

The 2D human keypoints provided in this dataset were generated through **manual frame-by-frame annotation**, rather than by a pretrained pose-estimation model or an automated keypoint-detection algorithm. For each video frame, the annotator visually inspected the participant’s body posture and manually identified the image locations of 12 major joints, including the left and right shoulders, elbows, wrists, hips, knees, and ankles. The annotated pixel coordinates were subsequently normalized by the image width and height to obtain two-dimensional coordinates ranging from 0 to 1. The resulting annotations were organized according to the corresponding frame sequence and stored in CSV format for each video.

## 5 Experimental Design, Materials and Methods

### 5.1 Experimental setup

This study employed a dual-camera synchronized recording design to comprehensively capture both frontal and lateral perspectives of the participant’s movements. Two cameras were mounted at identical heights and distances, positioned directly in front of and to the side of the subject, respectively, to record the entire sequence of Taekwondo Poomsae performances. The key experimental parameters are as follows:

• Camera height: Both cameras were mounted at 105 cm above ground level (lens center height ≈ 105 cm), ensuring full-body coverage of the subject (Fig. 5).

**Fig. 5:**
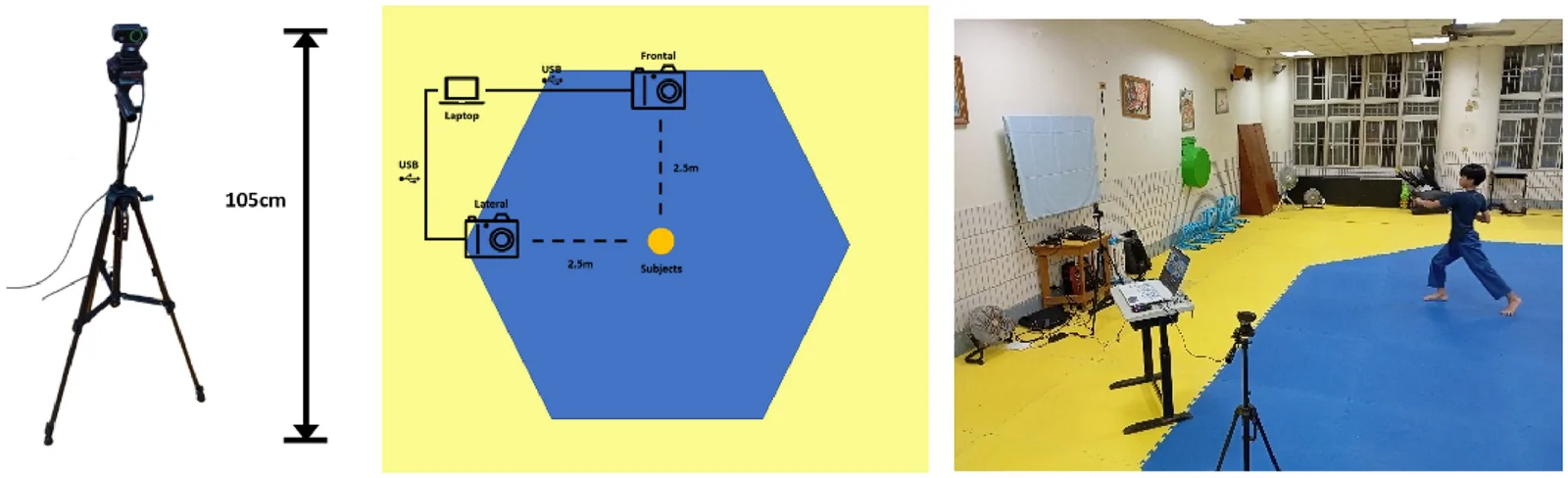
Illustration of experimental equipment and venue arrangement.
• Camera distance: The horizontal distance between the cameras and the subject was approximately 2.5 m. The subject stood at a pre-marked position to maintain consistent spacing across all recordings (Fig. 5).
• Camera placement: One camera was positioned directly in front of the subject (*Frontal* view), while the other was placed approximately 90° to the subject’s left side (*Lateral* view). The two cameras were aligned perpendicularly, enabling synchronized capture of movements from both perspectives (Fig. 5).

This arrangement ensured that dual-view synchronized recordings provided a more comprehensive representation of motion data, reducing the risk of joint occlusion or information loss that may occur in single-view recordings. During the experiment, participants performed the designated Taekwondo Poomsae sequences according to standardized instructions, while the camera system simultaneously captured the complete performance. All recordings were conducted in the same environment and under identical camera configurations to guarantee consistency and comparability of the collected dataset. The motor tasks performed in this study consisted of complete Taekwondo Poomsae routines from Taegeuk 1 to Taegeuk 8. Each Poomsae comprises a predefined sequence of offensive and defensive movements, including different stances and stepping movements, changes in body direction, upper-limb strikes and blocks, and lower-limb kicking techniques. The participant performed each Poomsae continuously according to its prescribed movement sequence rather than as isolated individual actions.

### 5.2 System architecture

The data acquisition system consisted of both hardware and software components, with the overall framework shown in Fig. 6. Two USB cameras were connected to a laptop computer, managed through a custom-designed graphical user interface (GUI) to enable synchronized dual-camera recording.

• Camera module: The system incorporated two cameras (*Frontal* and *Lateral*), dedicated to capturing frontal and lateral perspectives of the subject. Each camera produced video at a resolution of 1920 × 1080 (Full HD) and 30 fps, ensuring high-quality raw image data.
• GUI control module: A Python-based GUI (upper window in Fig. 6) provided recording status monitoring and control functions. The operator could simultaneously preview both video streams (lower preview windows in Fig. 6) and initiate or stop recording through button triggers. Upon receiving user input, the system dispatched synchronized control signals to both cameras, ensuring temporal alignment during recording.
• Data flow: Video streams from both cameras were transmitted via USB to the computer, where the GUI module managed the recording pipeline and wrote the synchronized frames to disk. When recording was initiated, both cameras started simultaneously; likewise, both terminated synchronously upon stopping. The resulting video files were temporally aligned, facilitating subsequent comparison and analysis.

**Fig. 6:**
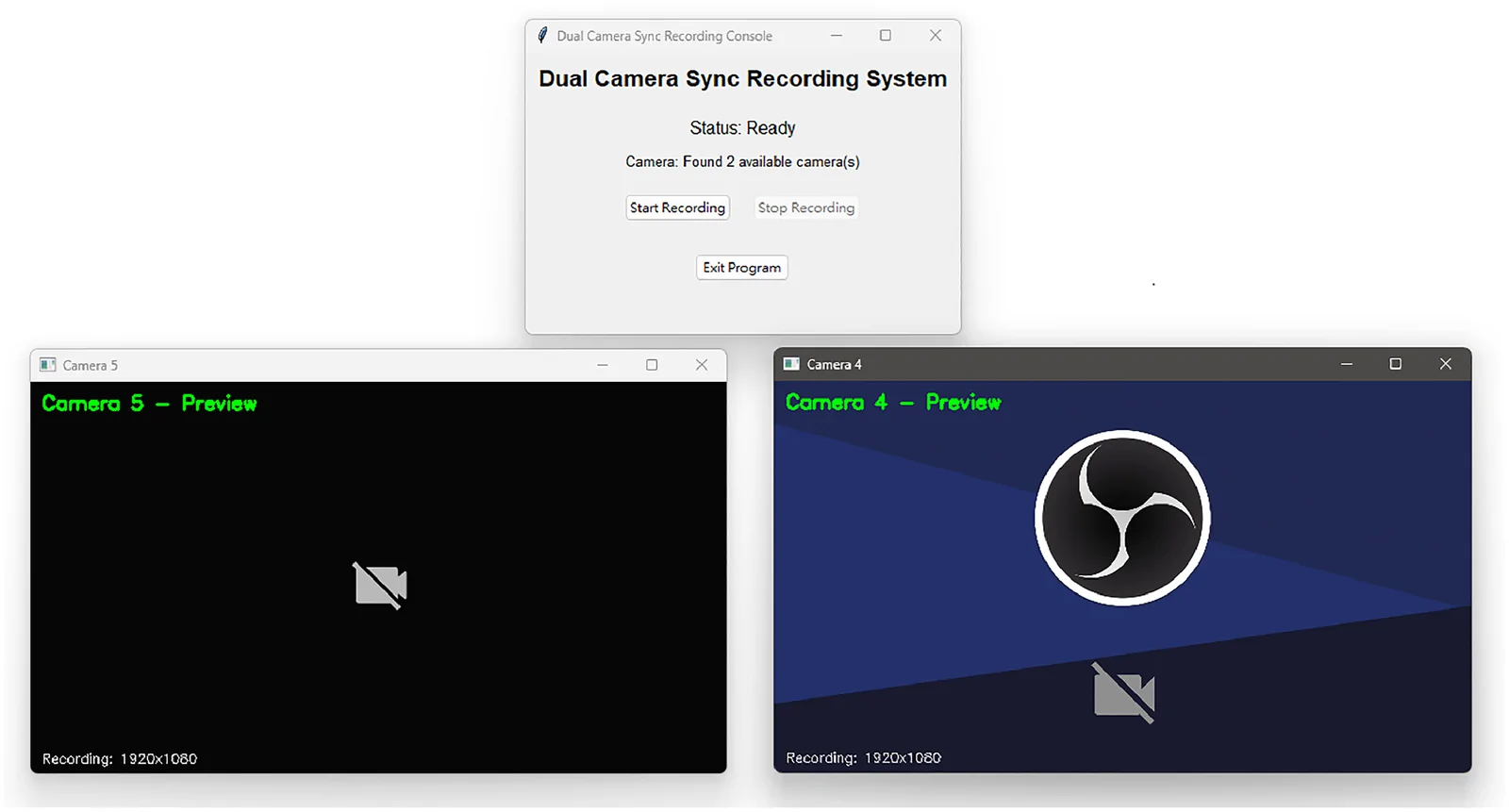
GUI interface for synchronized camera control.

### 5.3 Experimental procedure

To ensure high-quality and well-synchronized dual-view motion data, the cameras were pre-calibrated for position, angle, and focal length prior to formal recording. A fixed floor marker was used to guarantee that the participant consistently stood in the same location across trials.

The experimental procedure was standardized as follows: system initialization → preview calibration → participant positioning → synchronized recording start → participant completes routine → synchronized recording stop (Fig. 7). Throughout the entire process, both cameras were operated by a single GUI controller, thereby eliminating human-induced timing discrepancies.

**Fig. 7:**
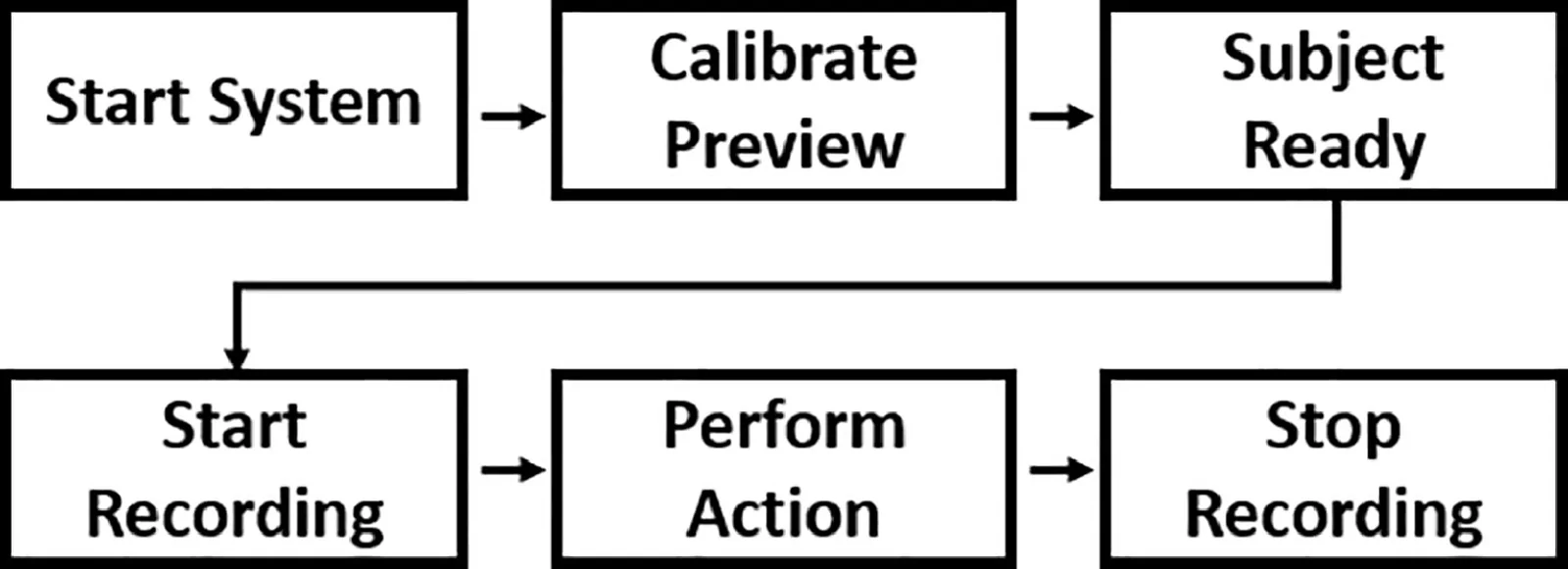
Workflow of synchronized camera operation.

## Limitations

This dataset was recorded at 30 fps with two fixed cameras (front and side) in a controlled indoor environment. Because standard (non-high-speed) cameras were used, fast motions (e.g., kicks) may exhibit motion blur/ghosting, which can affect the accuracy of some keypoints. We provide frame-level **2D** keypoint annotations only and did not apply additional deblurring/smoothing/interpolation on blurred frames.

Moreover, although two viewpoints are available, the dataset **does not include the calibration parameters required for 3D reconstruction** (intrinsics/distortion and the relative rotation/translation between cameras); consequently, reliable **3D** coordinates cannot be recovered from the provided files.

The dataset is suitable for **2D** pose analysis and method benchmarking of Taekwondo poomsae under consistent conditions; generalization to different environments/participants or tasks requiring high-speed capture and **3D** reconstruction may be limited.

## Ethics Statement

All data used in this study were collected with the participant’s prior informed consent and are accompanied by a signed written consent form (see attachment: Subject consent form.pdf), which has been archived. Before participation, the participant was fully informed of the study’s purpose, the methods of data collection and use, and the possibility of subsequent public sharing, and retained the right to freely decide whether to participate. All data are strictly limited to academic research and educational purposes and do not involve any commercial activities. This study complies with relevant research-ethics guidelines, and all signed consent documents have been securely stored to ensure transparency of the research process and to protect the participant’s rights.

## Credit Author Statement

**Huang, Chien-Chung:** Writing – original draft, Writing – review & editing, Validation, Methodology; **Zheng, Qing-Wei:** Data curation, Investigation, Visualization, Software.
